# Resonant Photoacoustic Spectroscopy of NO_2_ with a UV-LED Based Sensor †

**DOI:** 10.3390/s19030724

**Published:** 2019-02-11

**Authors:** Johannes Kapp, Christian Weber, Katrin Schmitt, Hans-Fridtjof Pernau, Jürgen Wöllenstein

**Affiliations:** 1 Department of Microsystems Engineering—IMTEK, University of Freiburg, 79110 Freiburg, Germany; christian.weber@imtek.uni-freiburg.de (C.W.); katrin.schmitt@imtek.uni-freiburg.de (K.S.); juergen.woellenstein@imtek.uni-freiburg.de (J.W.); 2 Fraunhofer Institute for Physical Measurement Techniques IPM, 79110 Freiburg, Germany; hans-fridtjof.pernau@ipm.fraunhofer.de

**Keywords:** resonant photoacoustic spectroscopy, LED, nitrogen dioxide (NO_2_) detection, T-cell

## Abstract

Nitrogen dioxide (NO_2_) is a poisonous trace gas that requires monitoring in urban areas. Accurate measurement in sub-ppm concentrations represents a wide application field for suitable economical sensors. We present a novel approach to measure NO_2_ with a photoacoustic sensor using a T-shaped resonance cell. An inexpensive UV-LED with a peak wavelength of 405 nm as radiation source as well as a commercial MEMS microphone for acoustic detection were used. In this work, a cell has been developed that enables a “non-contact” feedthrough of the divergent LED beam. Thus, unwanted background noise due to absorption on the inside walls is minimized. As part of the development, an acoustic simulation has been carried out to find the resonance frequencies and to visualize the resulting standing wave patterns in various geometries. The pressure amplitude was calculated for different shapes and sizes. A model iteratively optimized in this way forms the basis of a construction that was built for gas measurement by rapid prototyping methods. The real resonance frequencies were compared to the ones found in simulation. The limit of detection was determined in a nitrogen dioxide measurement to be 200 ppb (6 *σ*) for a cell made of aluminum.

## 1. Introduction

NO${}_{2}$ is a trace gas poisonous for humans, animals and plants. It is not only poisonous in itself but also the cause of summer smog (ozone). Due to the impact of sunlight, NO${}_{2}$ splits into likewise poisonous NO and a free oxygen radical. The latter forms ozone (O${}_{3}$) together with the atmospheric O${}_{2}$. The primary origins of NO${}_{2}$ are combustion processes of fossil fuels in motor vehicles and power plants [1]. The background concentration in the atmosphere is between 5 and 30 ppb [2]. However, close to its source it can be several magnitudes higher, thus being injurious to health [3]. Therefore, accurate measurements in the ppb range represent a wide application field, e.g., monitoring at inner-city traffic junctions, in tunnels and underground car parks [4]. To serve these claims, the sensor must not only be sensitive and selective, but also economical. So far, NO${}_{2}$ is measured by electrochemical sensors or large absorption photometers. Electrochemical sensors show a high sensitivity to NO${}_{2}$, are small and cost effective, but require a frequent replacement due to their limited lifespan. IR absorption photometers on the other hand need an absorption path of several meters to gain a sufficient sensitivity, which makes them very large and costly.

**Figure 1:**
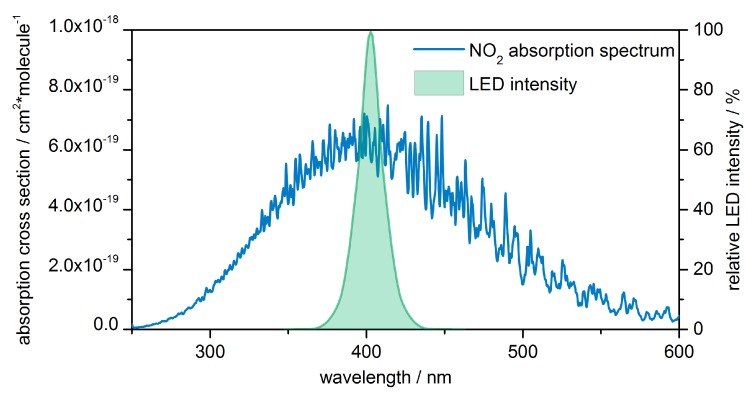
The emission spectrum of the used UV-LED with a peak wavelength of 405 nm. The datasheet states a spectral bandwidth of 15 nm [8]. The LED spectrum fits well to the absorption maximum of NO${}_{2}$ [9].

One of the most effective methods for trace gas measurement regarding sensitivity and selectivity is photoacoustic spectroscopy (PAS). The photoacoustic (PA) effect was discovered in 1880 by Alexander Graham Bell. The principle is based on the absorption of electromagnetic radiation by a medium of interest. The radiation energy is converted into heat. When using a pulsed radiation source, a thermal pressure wave is formed within the absorbing medium, which can then be sensed as sound by a microphone. The amplitude of the acoustic signal is directly proportional to the number of molecules in an absorbing gas which enables the determination of gas concentrations following proper calibration. However, due to the lack of appropriate instrumentation such as microphones, electronics and light sources, the PA effect was almost completely forgotten for more than half a century [5]. Gradually, with progress on the field of light sources, came new applications and possibilities on gas sensing via PAS. The technical developments of recent years have revealed powerful LEDs for a wide range of wavelengths. These are highly compact, robust, efficient and inexpensive compared to lasers. Especially UV-LEDs fit the absorption spectrum of NO${}_{2}$ well (see Figure 1), making them the light source of choice for our approach. UV-LEDs have already been used for PAS of NO${}_{2}$ by Santiago et al. in 2006, achieving a limit of detection (LOD) of 5 ppm (3 σ) [6]. Five years later, Saarela et al. presented their LED setup with a LOD of 10 ppb with a signal to noise ratio (SNR) of one, though with limitations regarding long-term measurement due to heat-up of the cell by the used LED array [7].

**Figure 2:**
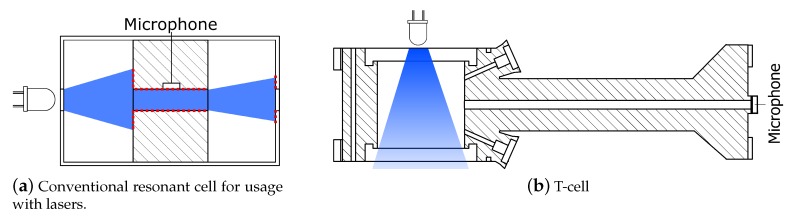
(**a**) Schematic drawing: Diverging LED light within a non-optimized resonant cell similar to that proposed in Ref. [5]. The light hits the inner walls at the red dashed line, causing the background signal. The lower signal to offset ratio results in a worse limit of detection and stability problems. (**b**) Concept for use of LED light. The T-cell consists of an absorption chamber that enables the non-contact feedthrough of the beam. Perpendicular to it there is the resonance cylinder, where the standing wave pattern is formed.

PA measurement is usually performed within a closed cell. The cavity can be used for resonant amplification. Various cell designs for usage with lasers have been proposed and investigated by Miklós and Hess [5]. Due to the limited focusing and collimating capability of LEDs, however, in conventional cell designs the light hits the inner walls with high probability (outlined in Figure 2a). There, it absorbs as well and generates unwanted noise (interfering signal offset), which results in a worse LOD. Lassen et al. [10] followed the approach of using an integrating sphere with a high reflectance for signal enhancement and the attenuation of the background absorption. Their achieved LOD is 1.9 ppm with a SNR of one.

The main focus of this work is the reduction of the background signal by minimizing the light incidence on the inside walls of the PA cell. This is achieved by using a concept named T-cell. It is derived from a geometry that consists of an absorption chamber and a perpendicular resonance cylinder, where the standing wave pattern is formed (see Figure 2b). The resonator has a pressure node at the joint to the absorption chamber and a pressure maximum, where the microphone is mounted. Up till now, T-cells have been used with lasers, e.g., by Baumann et al. 2005 [11] and Liu et al. 2006 [12], yet these cell designs are not suited for LEDs. We present a cell where the dimensions are defined in a way that a non-contact feedthrough of the LED beam is possible. Furthermore, they were iteratively optimized by finite elements method (FEM) simulations using ANSYS Workbench. A constructed prototype was fabricated from aluminum by rapid prototyping methods. The sensor was characterized by gas measurements. The present paper is an extended version of work published in Ref. [13].

## 2. Design and Methods

### 2.1. Sensor Design

**Figure 3:**
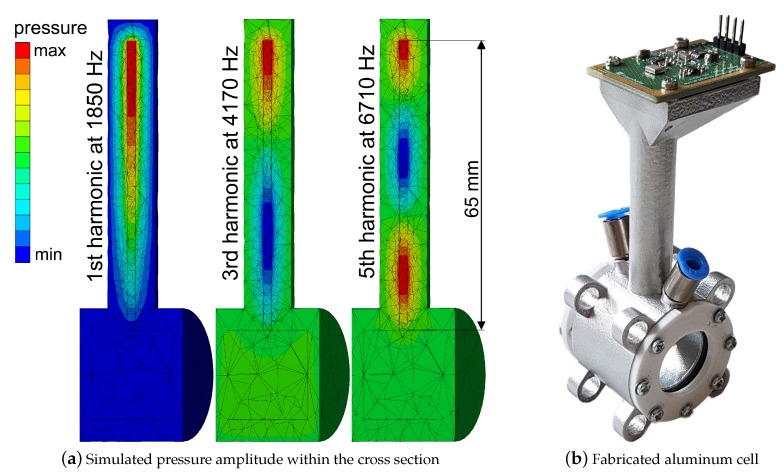
(**a**) Cross section of the cell with simulated pressure amplitude corresponding to standing wave patterns of different harmonics. One can observe the first harmonic at 1850 Hz, the third harmonic at 4170 Hz and the fifth at 6710 Hz. Pressure peaks (red) are at the spot, where the microphone will be assembled. As expected, no standing wave pattern is formed within the absorption chamber. (**b**) Picture of the aluminum cell with mounted microphone and windows (100 mm × 40 mm × 40 mm).

For acoustic simulation, the FEM software ANSYS Workbench 19.0 was used. We used a combination of the two acoustic modules “modal acoustics” (MA) and “harmonic acoustics” (HA). MA determines the eigenmodes of a certain structure, whereas HA analyses its stationary answer to a predefined excitation. The solution includes the spatial pressure amplitude and its gradient. A 3D-CAD model of the cell was created, which consists of three components: air within the cell (cavity), the cell itself and the ambient air. The T-cell is subdivided into the absorption chamber and the resonance cylinder. Considering their dimensions: On the one hand, the absorption chamber diameter has to be large enough for the feedthrough of the LED beam. On the other hand, the PA signal is inversely proportional to the modulation frequency and the volume of the cell [5]. Furthermore, we wanted to avoid the formation of a standing wave within the absorption chamber since there are the less reverberant windows and gas in- and outlets, potentially coupling energy of the standing wave out of the system. Therefore the dimensions of the absorption chamber have to be small compared to the wavelength of the excitation frequency. As a compromise, both diameter and length are set to 20 mm. The resonance frequency is predefined by the length of the resonance cylinder. As noise sources, e.g., intrinsic noise of the microphone, amplifier noise and external acoustic noise, show a characteristic 1/*f* frequency dependence, it can be advantageous for the SNR to apply high excitation frequencies [5]. The source of excitation in simulation was a punctual monopole with a radius of 3 mm and an amplitude of 1 Pa, placed within the middle of the absorption chamber. Materials are assigned to aluminum for the cell itself as well as air within and outside, with material parameters from ANSYS engineering data. Wall thickness of the cell is uniformly 5 mm. The result of the simulation is shown in Figure 3a. The observed patterns correspond to a system with an open and a closed end accordingly, with no standing wave within the absorption chamber. Pressure peaks are located at the microphone spot for all three modes. One can observe that parts of the pressure are conducted within the material of the cell. This illustrates the influence of the speed of sound within the solid body onto the resulting resonance frequency.

**Figure 4:**
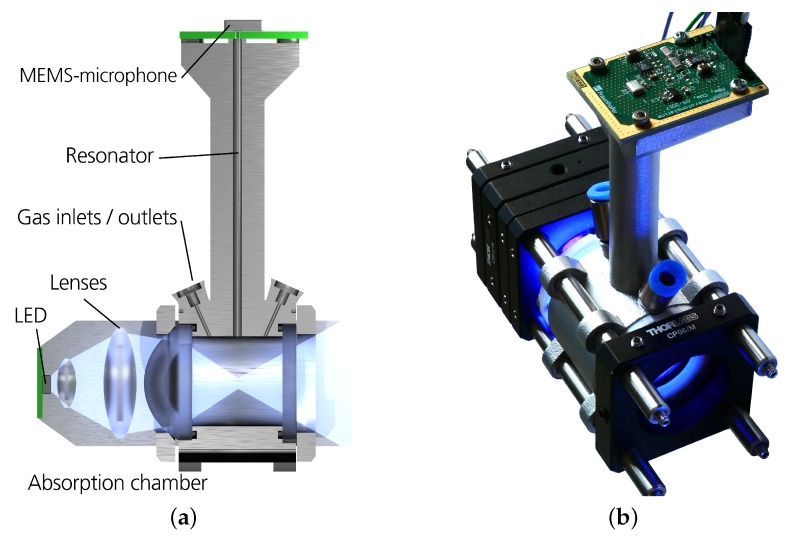
(**a**) Sensor setup in principle. (**b**) Picture of the setup with optical components mounted in a Thorlabs cage system (100 mm × 40 mm × 750 mm).

Based on the simulation results, an absorption chamber diameter of 20 mm and a length of 20 mm were chosen for our sensor setup. With a length of 65 mm, the resonator is designed to have its third mode at approximately 4 kHz. The cell fabricated out of aluminum via SLM (selective laser melting) is shown in Figure 3b. A 15335340AA350 type LED by Würth Electronic is used as light source. It has a peak wavelength of 405 nm with a spectral bandwidth of 15 nm (as shown in Figure 1) and is driven by a sinusoidal current with 200 mA peak [8]. The resulting low peak power allows a passive cooling of the LED. A combination of three lenses focuses the light into the cell (see Figure 4): one with 15 mm and two with 25.4 mm focal length. The used microphone is a bottom port microphone (ICS-40720, TDK, Tokyo, Japan). It was soldered onto a printed circuit board that was mounted at the closed end of the resonator. The microphone signal is preamplified, AD-converted and digitally processed on a custom board, that also generates the signal to modulate the LED current. The captured microphone signal is filtered by a digital lock-in algorithm with an integration time of 1.14 s, and transmitted to a PC.

### 2.2. NO${}_{2}$ Gas Measurements

To determine the frequency response of the resonant cell and the gas response of the sensor, gas measurements were performed. The frequency response of the resonant cell was measured by applying a permanent flow of 22.5 ppm NO${}_{2}$ at 0.1 L/min through the cell, while sweeping the LED modulation frequency from 3 kHz to 10 kHz covering the frequencies of the 3rd to 7th harmonic. The frequency range below 3 kHz was not included because the cutoff frequency of the high pass filter was set to 2.5 kHz in order to reduce the overall 1/*f* noise. To determine the LOD, the cell was perfused with alternating steps of 100% nitrogen and different NO${}_{2}$ (in synthetic air) concentrations from 850 ppb to 4.3 ppm. The uncertainty of the NO${}_{2}$ setpoints is maximum 12%, originating from massflow controller and test gas uncertainties. Each concentration step was measured for 30 min. With these concentrations in the cell, the modulation frequency of the LED is swept just over the frequency range of one resonance peak (3.5 to 3.9 kHz covering the third harmonic of the resonator). Every single frequency point in the sweep is sampled for around 1.14 s, which corresponds to the lock-in integration time. The sweep data is evaluated using a simple algorithm that selects a three point average around the highest point of every sweep as a representative value. This approach eliminates the need for active resonance tracking on the cost of a much lower data rate. All captured data points are lock-in raw data without any compensation or calibration applied. To determine the zero point stability, the sensor was operated for 72 h in ambient air with open in- and outlets. The LED excitation frequency in this case was fixed at 3.80 kHz, which corresponds to the resonance maximum of the third harmonic in air. The lock-in integration time was still 1.14 s.

## 3. Results and Discussion

**Figure 5:**
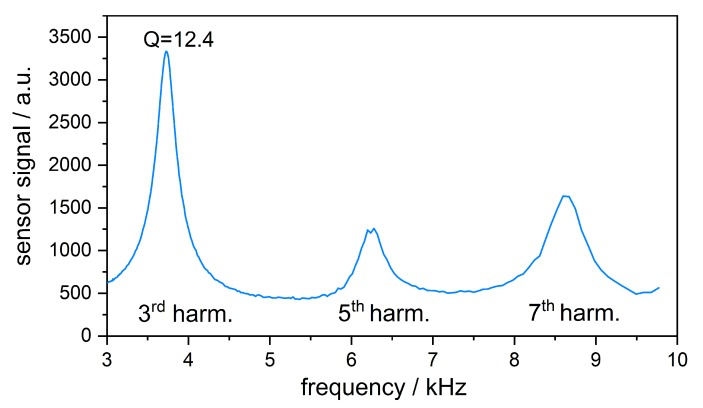
Frequency response of the resonant photoacoustic cell with peaks of different harmonics: 3rd at 3727 Hz, 5th at 6275 Hz, 7th at 8635 Hz. The 3rd harmonic gives the best signal and has a Q-factor of around 12.4.

Figure 5 shows the results of the frequency response measurement. The third harmonic is with 3.7 kHz close to the design target of 4 kHz. Compared to the modes found in simulation, the measured ones are lower due to the extended resonator length caused by the microphone mounting. Besides this shift, the other modes are also close to the predicted frequencies. The fifth mode, however, amplifies the signal less than expected.

The third harmonic gives the best signal amplification with a Q-factor of 12.4. Due to the high quality factor, the third harmonic was chosen as frequency range for the following gas measurements. Despite the fact that the PA effect scales inversely proportional with the modulation frequency we found that the first harmonic could hardly be excited, independent of the electrical high pass cutoff frequency.

**Figure 6:**
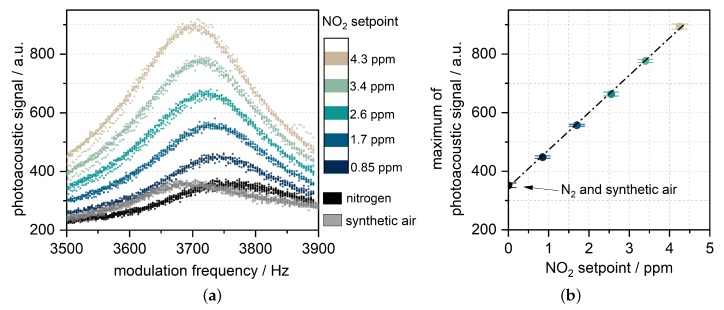
(**a**) Lock-in filtered microphone signal (integration time of 1.14 s) while sweeping the LED modulation frequency over the third harmonic of the resonator at different NO${}_{2}$ concentrations. A reading is taken every 0.75 s. The resonance frequency shift is due to the dilution of NO${}_{2}$ in synthetic air. (**b**) Mean value of the three highest points of the sweep, together with their standard deviation (±1σ) and the linear correlation.

Figure 6a shows the photoacoustic signal during the frequency sweeps for different concentrations of NO${}_{2}$. As expected, the resonance amplitude rises with increasing NO${}_{2}$ concentration. Once a concentration step is established, the sweeps are almost congruent to each other. Because NO${}_{2}$ was diluted in synthetic air, the contained oxygen (20% per 4.3 ppm NO${}_{2}$) caused a resonance frequency shift of up to 60 Hz. This value is consistent with the theoretical shift due to change in speed of sound of the gas matrix. Without NO${}_{2}$, the oxygen concentration had no influence on the signal amplitude (also shown in Figure 6a). The maximum value of every sweep was evaluated within the data processing of the custom board. Figure 6b shows these values plotted against the NO${}_{2}$ setpoint. It can be observed that the relation is linear as expected. The sensitivity can be determined by the slope of the graph which is around 125 counts per ppm. From the standard deviation shown in Figure 6b and the sensitivity, a noise equivalent concentration of 32 ppb (1 σ) is calculated. This results in a LOD at around 200 ppb (6 σ).

**Figure 7:**
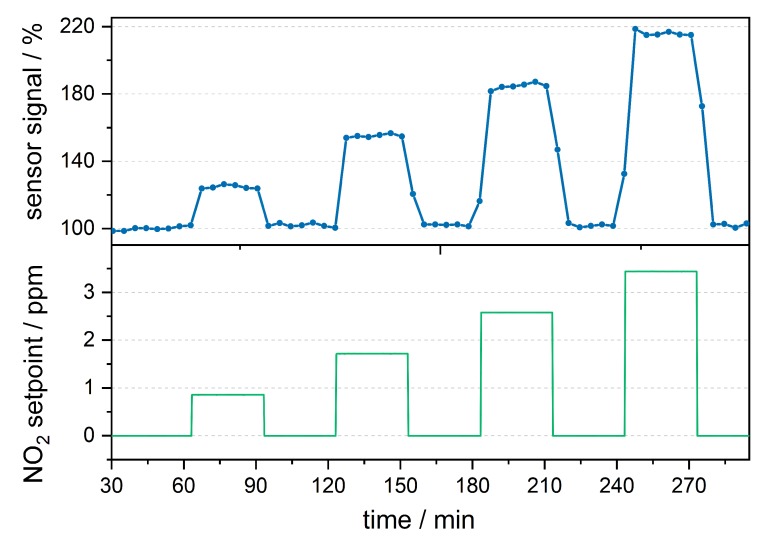
Time resolved gas measurement result of the photoacoustic sensor normalized to the zero concentration. Due to the sweep over the complete resonance peak the data rate is around 0.25 min${}^{-1}$. The noise analysis showed a noise equivalent (1 σ) of approximately 32 ppb. Therefore the sensor can detect concentrations of around 200 ppb with 6 σ confidence.

**Figure A1:**
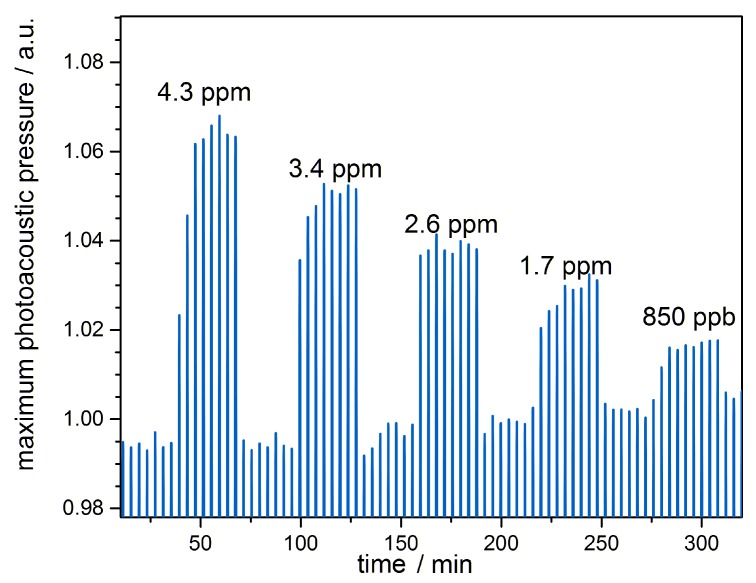
Measurement with the same setup but with a H-cell similar to the work of El-Safoury et al. [14]. The H-cell has a resonator length of 40 mm and a diameter of 3 mm. The buffer volumina have both a diameter and a length of 20 mm. In this setup, the offset is equivalent to 65.4 ppm NO${}_{2}$. Due to the high offset, the zero gas signal is more vulnerable to drift effects and therefore less stable.

Figure 7 shows the processed data plotted against the time, together with the NO${}_{2}$ concentration setpoint. Due to residual stray light, there is a zero gas offset of 370 counts which is normalized to 100%. The signal offset is equivalent to 2.88 ppm. This is significantly lower than the offset equivalent to 65.4 ppm, that was observed in an H-cell with the same setup as shown in Figure A1. Due to the small offset, there is no observable drift in the zero signal during the measurement time of five hours. The data rate of the measurement was low (0.25 min${}^{-1}$) due to the simple algorithm choosing only one data point per sweep. With an intelligent tracking algorithm, the data rate could be increased to 1–10 s${}^{-1}$ without any loss of resolution. Although there is photodissociation of NO${}_{2}$ below 415 nm, the presented measurement does not seem to be affected by this dissociation, due to continuous gas flow [7].

**Figure 8:**
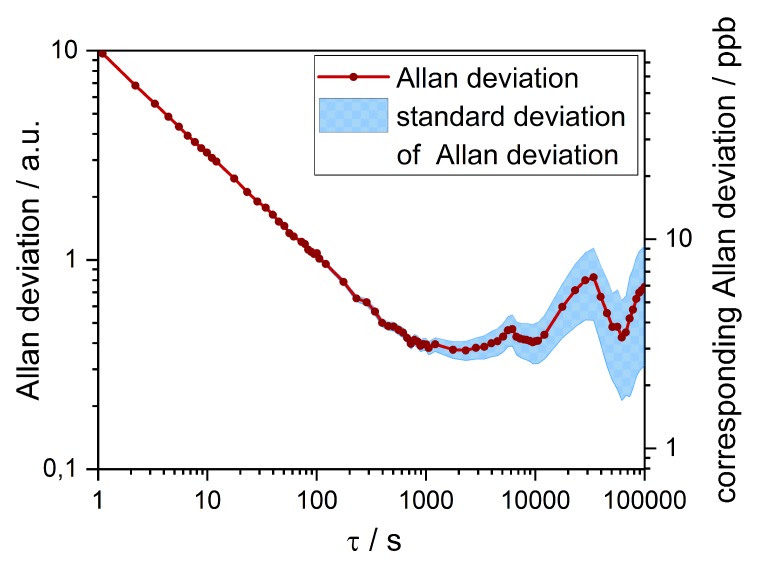
Calculated Allan deviation of the ambient air signal over a measuring time of 72 h. The left axis shows the Allan deviation in counts. On the right axis it is converted into ppb using the sensors sensitivity of 125 counts per ppm.

The data from the zero point stability measurement is evaluated in an Allan plot that is shown in Figure 8. For one second of averaging time, the Allan deviation is around 10 counts which translates into 80 ppb of NO${}_{2}$. For longer averaging times of up to 1000 s, this value drops to 3 ppb. For longer intervals the Allan deviation rises again but stays below 10 ppb. The reason for this could be fluctuations in ambient temperature, atmospheric NO${}_{2}$ or the resonance frequency.

## 4. Conclusions

We have shown a novel approach for highly sensitive NO${}_{2}$ detection using a photoacoustic sensor operating in the UV. This sensor combines for the first time the advantages of low-cost components (UV-LED, commercial MEMS microphone) with a T-shaped cell fabricated in rapid prototyping. The T-shaped cell enables a “non-contact” feedthrough of the divergent LED beam in the absorption cylinder, leading to a significant reduction of background signal and thus higher sensitivity. The PA cell was first simulated in ANSYS to find optimal geometries for absorption and resonance cell, and the spatial pressure amplitude and its gradient. After fabrication of the cell in aluminum and setup of the complete sensor system, this was characterized with NO${}_{2}$ concentrations ranging from 850 ppb to 4.3 ppm. From this measurement, a LOD of around 200 ppb (6 σ) could be deduced. Furthermore, the results show no observable drift in the zero signal for at least five hours.

Our study shows the possibility of reliably measuring NO${}_{2}$ in concentrations well below 1 ppm using a compact photoacoustic setup (100 mm × 40 mm × 750 mm) with commercially available, low-cost components. This makes the sensor an alternative to the commonly used electrochemical cells for NO${}_{2}$ monitoring. Future work will comprise an advanced resonator design that might allow even higher resolution while enabling a further miniaturization of the sensor. Furthermore, we plan a more detailed study on the reproducibility and repeatability of the sensor system.

## Abbreviations

The following abbreviations are used in this manuscript:

ppm	parts per million
ppb	parts per billion
PA	photoacoustic
PAS	photoacoustic spectroscopy
LOD	limit of detection
FEM	finite elements method
MA	modal acoustics
HA	harmonic acoustics
SNR	signal to noise ratio
